# A mapping and synthesis of tools for stakeholder and community engagement in quality improvement initiatives for reproductive, maternal, newborn, child and adolescent health

**DOI:** 10.1111/hex.13237

**Published:** 2021-04-01

**Authors:** Jessie Spencer, Brynne Gilmore, Elsbet Lodenstein, Anayda Portela

**Affiliations:** ^1^ Division of Public Health Michigan State University College of Human Medicine Flint MI USA; ^2^ UCD Centre for Interdisciplinary Research Education and Innovation in Health Systems School of Nursing, Midwifery and Health Systems University College Dublin Dublin Ireland; ^3^ KIT Royal Tropical Institute Amsterdam the Netherlands; ^4^ Department of Maternal, Newborn, Child and Adolescent Health and Ageing World Health Organization Geneva Switzerland

**Keywords:** child and adolescent health, maternal, newborn, quality improvement, quality of care, stakeholder and community engagement

## Abstract

**Background:**

Stakeholder and community engagement promotes collaboration and gives service users an opportunity to actively participate in the care they receive. Recognizing this potential, The Network for Improving Quality of Care for maternal, newborn and child health aimed to identify tools and operational guidance to integrate stakeholder and community engagement into quality improvement (QI) implementation.

**Methods:**

A mapping, consisting of a literature review and an open call through email and listservers, for implementation tools was conducted. Materials were included if they provided guidance on stakeholder and community engagement aligned to the Network's QI framework comprising seven phases. Screening of tools was done by two reviewers.

**Results:**

The literature search and the call for tools returned 197 documents with 70 tools included after screening. Most included tools (70%) were published after 2010. International organizations were the most frequently cited authors of tools. Only 15 tools covered all seven phases of the QI framework; few tools covered the more ‘technical’ phase of the QI framework: adapting standards and refining strategies.

**Conclusion:**

The quantity of tools and their varied characteristics including types of stakeholder and community engagement processes across the QI framework confirms that engagement cannot be captured in a ‘one‐size‐fits‐all’ formula. Many tools were designed with a generic focus to allow for adaption and use in different settings and sectors. Country programmes looking to strengthen engagement approaches can take advantage of available tools through an online portal on the WHO website and adapt them to meet their specific needs and context.

**Public Involvement:**

Programme implementers provided tools and resources during data collection.

## 1 INTRODUCTION

Stakeholder and community engagement is widely advocated for within health programming and is of paramount importance to achieving universal health coverage^1^, ^2^ and primary health care for all.^3^, ^4^ Defined as a process of involving communities in decision‐making and in the planning, design, governance and delivery of service,^5^ stakeholder and community engagement promotes collaborative relationships between stakeholders (i.e. community members, patients, health personnel, local authorities, policymakers and non‐governmental organizations) and gives service users an opportunity to be active participants in the care they receive.

Although stakeholders and community members are frequently consulted in health programming, their engagement has been underdeveloped in efforts to improve the quality of health services provided (herein referred to as quality improvement (QI) initiatives), as historically this has been the responsibility of health professionals, managers and planners. Non‐health stakeholders, particularly community representatives, are rarely seen as knowledgeable enough to participate in QI initiatives, and when included, their engagement is often limited to providing input into problem identification or serving as (unpaid) human resources for health promotion and programme implementation.^6^, ^7^

Principles of stakeholder and community engagement are entrenched in Alma Ata's call for full participation of individuals and communities in health care^8^ and are aligned to the fundamental right of individuals to be engaged in processes surrounding their health needs and decisions.^9^ Stakeholder and community engagement interventions have proven effective in improving health behaviours and outcomes,^4^, ^5^, ^10^, ^11^ providing more acceptable and people‐centred services^3^, ^12^ and empowering communities, resulting in transformative change beyond the health sphere.^6^ Benefits of stakeholder and community engagement for quality of care has been noted across various stages of QI initiatives including design and planning,^13^ implementation and delivery,^14^ monitoring and accountability^15^ and for building relationships between providers and clients.^16^ For these reasons, stakeholder and community engagement has been identified as an integral component of improving the quality of care for women, newborns and children, as participatory approaches encourage relevant, effective, context‐specific interventions^17^, ^18^, ^19^ and can hold service providers and policymakers accountable for quality and implementation.^15^, ^20^ Numerous frameworks and reviews exist to provide theoretical and conceptual considerations in guiding the design and implementation of stakeholder and community engagement programmes (Box 1). They highlight the increasing demand for stakeholder and community engagement to be embedded into health‐care strategies and QI initiatives.

However, stakeholder and community engagement is not universally embraced. A recent World Health Organization (WHO) policy survey across all six geographic regions found that only 60% of countries have implemented mechanisms at the facility level to solicit feedback on quality and access from community members, users and families. The response varied by region, ranging from a high of 72.7% in countries in the South‐East Asian region, 69.2% in the European region, 62.5% in the Eastern Mediterranean region, 58.5% in the African region, 53.3% in the American region and 47.1% in the Western Pacific region.^21^

BOX 1 **Selected frameworks, toolkits and reviews for stakeholder and community engagement**

**Frameworks**

- A framework for community engagement in primary health. Saskatoon Health Region, Primary Health and Chronic Disease Management. Saskatoon, 2012.
- CAMH Community Engagement Framework. Centre for Addiction and Mental Health, A Pan American Health Organization/World Health Organization Collaborating Centre
- Community engagement: improving health and well‐being and reducing health inequalities: NICE guideline. United Kingdom: NICE, 2016
- LHIN Community Engagement Guidelines and Toolkits. Ontario, Canada: Ministry of Health and Long‐Term Care, Ontario, 2011.
- The National Standards for Community Engagement 2015/2016, Communities Scotland, Scottish Executive. 2016
- WHO Community Engagement Framework for Quality, People‐Centred and Resilient Health Services. Geneva: World Health Organization, 2017.
- WHO, Enfants du Monde, PAHO. Working with individuals, families and communities to improve maternal and newborn health: a toolkit for implementation. Geneva: World Health Organization,, 2017

**Reviews**

- Brunton G, Thomas J, O’Mara‐Eves A, Jamal F, Oliver S, Kavanagh J., 2017. Narratives of community engagement: a systematic review‐derived conceptual framework for public health interventions. *BMC public health, 17*(1):944.
- Cyril S, Smith BJ, Possamai‐Inesedy A, Renzaho AMN., 2015. Exploring the role of community engagement in improving the health of disadvantaged populations: a systematic review. *Global Health Action, 8*(1):29842.
- De Weger E, Van Vooren N, Luijkx K, Baan C, Drewes H. 2018 Achieving successful community engagement: a rapid realist review. *BMC health services research, 18*(1):285.
- George AS, Branchini C. 2017. Principles and processes behind promoting awareness of rights for quality maternal care services: a synthesis of stakeholder experiences and implementation factors. BMC Pregnancy Childbirth 17(264)
- Lodenstein E, Dieleman M, Gerretsen B, Broerse JE., 2017. Health provider responsiveness to social accountability initiatives in low‐ and middle‐income countries: a realist review. *Health Policy Planning, 32*(1):125‐40.
- Martin Hilber A, Blake C, Bohle LF, Bandali S, Agbon E, Hulton L., 2016. Strengthening accountability for improved maternal and newborn health: A mapping of studies in Sub‐Saharan Africa. *International Journal of Gynecology & Obstetrics, 135*(3):345‐57.
- McCoy, DC, Hall, JA and Ridge, M., 2011. A systematic review of the literature for evidence on health facility committees in low‐and middle‐income countries. *Health policy and planning, 27*(6), pp. 449‐466.
- Molina, E., Carella, L., Pacheco, A., Cruces, G. and Gasparini, L., 2016. Community monitoring interventions to curb corruption and increase access and quality of service delivery in low‐and middle‐income countries: a systematic review. *Campbell Systematic Reviews, 12*.
- Molyneux, S., Atela, M., Angweni, V. and Goodman, C. 2012. Community accountability at peripheral health facilities: a review of the empirical literature and development of a conceptual framework. *Health policy and planning, 27* (7), pp. 541‐554.
- Mubyazi G, M., Guy H., 2012. Rhetoric and reality of community participation in health planning, resource allocation and service delivery: a review of the reviews, primary publications and grey literature. *Rwanda Journal of Health Sciences, 1*(1):51‐65.
- O'Mara‐Eves A, Brunton G, McDaid D, Oliver S, Kavanagh J, Jamal F, et al, 2013. Community engagement to reduce inequalities in health: a systematic review, meta‐analysis and economic analysis. Public Health Research. Southampton (UK)
- Rifkin, SB, 2009. Lessons from community participation in health programmes: a review of the post‐Alma‐Ata experience. *International Health*, *1*(1), pp. 31‐66.
- Sacks E, Swanson RC, Schensul JJ, Gleave A, Shelley KD, Were MK, et al, 2017 Community involvement in health systems strengthening to improve Global Health outcomes: a review of guidelines and potential roles. *International quarterly of community health education, 37*(3‐4):139‐49.
- Swainston K, Summerbell C. The effectiveness of community engagement approaches and methods for health promotion interventions. Rapid Review. Phase 3 (including consideration of additional evidence from stakeholders), 2008. Teesside: NICE National Collaborating Centre University of Teesside.

The importance of quality of care, and thus an emphasized need for stakeholder and community engagement to improve quality, is especially pertinent within the field of reproductive, maternal, Newborn, Child and Adolescent Health (RMNCAH). While access to care has increased, reductions in maternal and child mortality have not followed suite with poor quality of care being highlighted as a main driver of such inequities.^22^ Poor quality of services for women and children is well documented, especially from low‐ and middle‐income countries^23^ and has been found to influence utilization^24^ and outcomes^25^ of health care.

To this end, The Network for Improving Quality of Care for maternal, newborn and child health (the Network; Box 2) envisions quality of care in terms of the provision of care, the way it is delivered by health workers, and experience of care by women, newborns, children and families.^19^, ^26^

BOX 2 **The Network for Improving Quality of Care for maternal, newborn and child health (MNCH)**

The Network, supported by WHO and UNICEF, consists of 10 countries who are committed to achieving a vision where every pregnant woman and newborn infant receives quality care throughout pregnancy, childbirth and the postnatal period. Currently, ten countries across sub‐Saharan Africa and South Asia have joined the Network: Bangladesh, Côte d’Ivoire, Ethiopia, Ghana, India, Malawi, Nigeria, Sierra Leone, United Republic of Tanzania and Uganda.

**What are the goals of the Network?**

- Reduce maternal and newborn mortality in health facilities in target country districts by 50% over 5 years and to halve intra‐partum stillbirths;
- Reduce avoidable morbidity targeting a 50% reduction in severe post‐partum haemorrhage and of neonatal sepsis;
- Improve experience of care.

**What will the Network do?**

- Focus on national leadership by strengthening national and district governance quality of care structures and helping develop national plans and advocacy strategies for improving quality of care.
- Accelerate action by adapting and adopting WHO’s eight Standards for improving quality of maternal and newborn care in health facilities at country level, creating national packages of quality improvement interventions and develop, strengthen and sustain clinical and managerial capabilities to support quality of care improvement.
- Foster learning and generate evidence on quality of care through a Learning Platform—a community of health practitioners from around the world co‐developing and sharing knowledge, country data and research to inform maternal and newborn quality of care improvement work in countries. The Learning Platform's outcomes will feed into the WHO‐led Global Learning Laboratory for Quality Universal Health Coverage.
- Develop and support institutions and mechanisms for accountability for quality of care by designing a national accountability framework and monitoring the progress of the Network for improving quality of care for MNCH.

**FIGURE 1:**
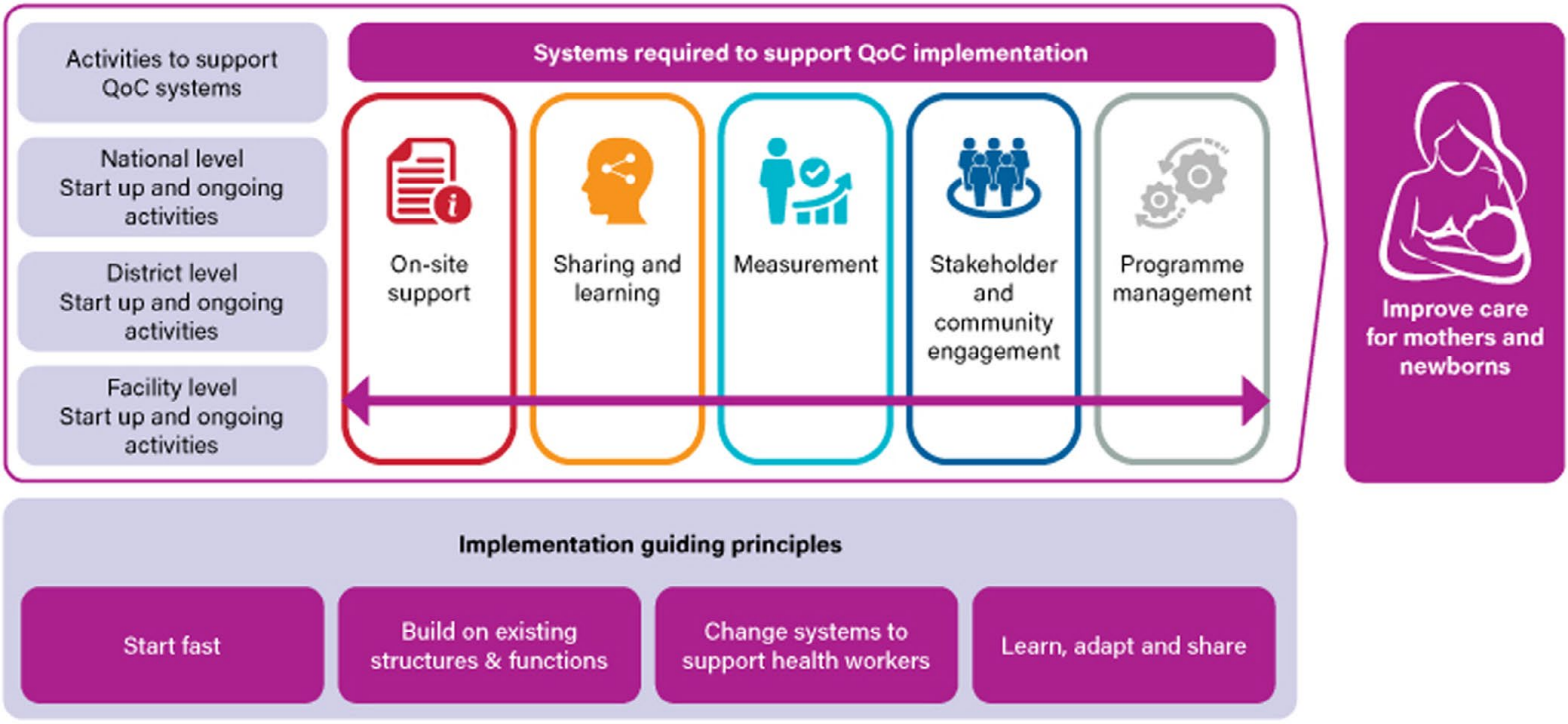
Quality of Care Implementation guide components. Adapted from Ref. [27]

The Network has included stakeholder and community engagement within its implementation guidance as a core system required to support QI implementation (Figure 1).^27^

**FIGURE 2:**
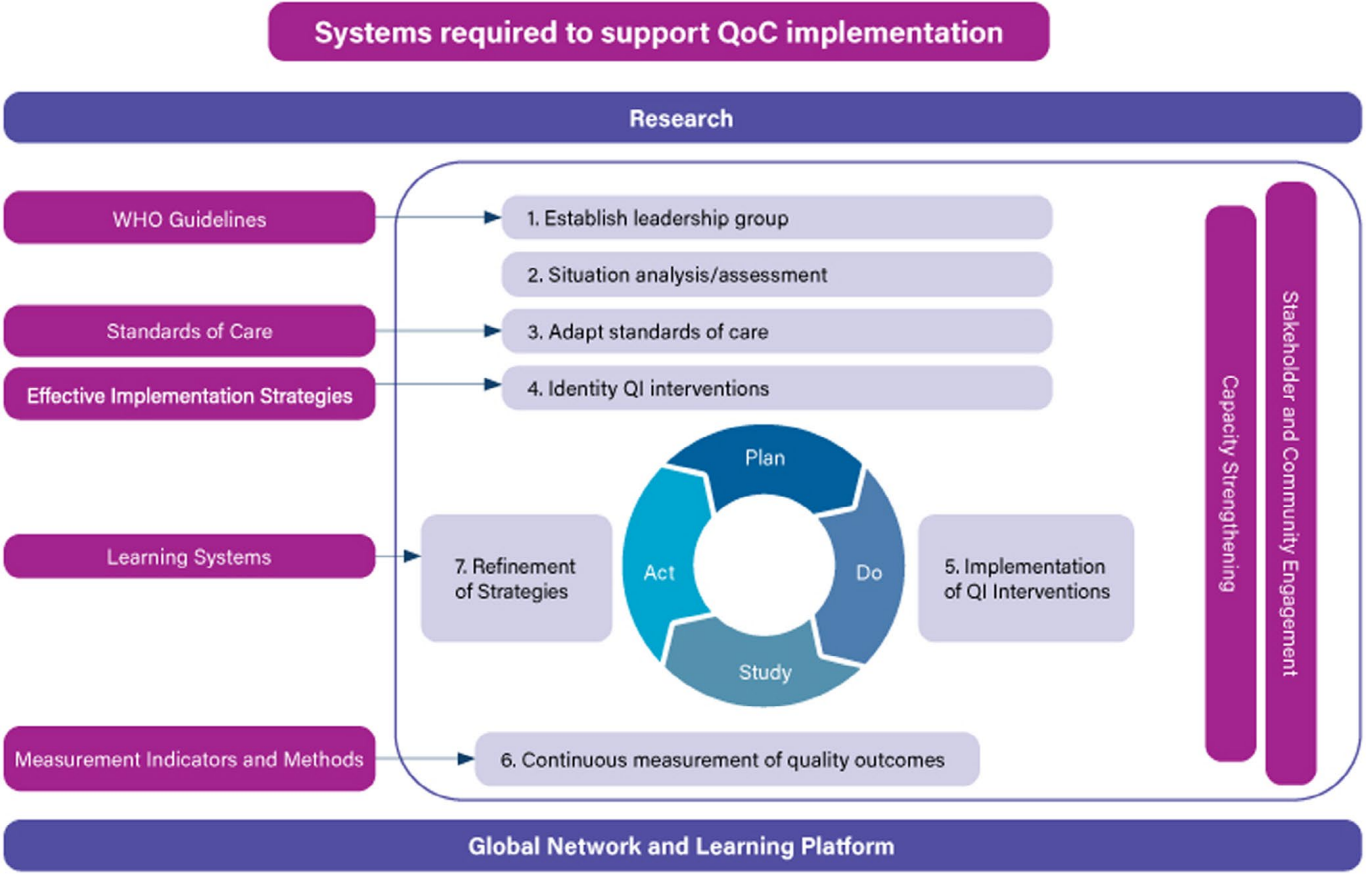
The Network's Implementation framework for improving quality of care for maternal, newborn and child health^28^

Feedback from countries in the Network indicated interest in receiving support on how to integrate stakeholder and community engagement into QI implementation. In order to respond to this need and to build on existing efforts, we conducted a mapping exercise to identify and compile existing tools on stakeholder and community engagement and link them to the seven different phases of the QI implementation framework (Figure 2). The results of the mapping informed the development of a module integrated into the Network's implementation guidance. This paper presents the methods and results of the mapping exercise and outlines gaps and opportunities for future tool development and support.

## 2 METHODS

A mapping exercise was conducted to identify and synthesize relevant tools (i.e. formal documents, handbooks and training guides) that could inform the implementation of stakeholder and community engagement across the seven phases of the QI implementation framework (Figure 2). The mapping consisted of a two‐stage document search. Stage one consisted of a literature search which identified published guidelines, frameworks and literature reviews, which was used to identify relevant programmes and organizations who developed tools. Stage two involved a call through email and listservs requesting tools from partner agencies and academic institutions, contacting relevant authors or organizations identified during stage one, and snowballing.

### 2.1 Stage 1: literature search

From December 2017 to July 2018, an iterative literature search was conducted to gather reviews, frameworks and guidelines that provided details or implementation support on stakeholder and community engagement in QI initiatives for health. We searched two academic databases (PubMed and EMBASE) using MESH search terms ‘community engagement’ or ‘social accountability’ or ‘stakeholder engagement’ and ‘maternal and child health‘ and ‘tools’ or ‘frameworks’ or ‘guides’ or ‘handbooks', to identify any academic resources. Grey literature was also identified through WHOLIS, extensive Google searches and reviews of relevant organizational websites. Additional relevant literature was identified through an evidence map on social, behavioural and community engagement (SBCE) interventions for reproductive, maternal, newborn and child health.^29^ Searches were conducted in English, no language restrictions were applied, and dates were restricted to 2010 to present. Identified resources were screened to find programmes or mentions of tools that could be relevant to the mapping.

### 2.2 Stage 2: open call for tools

The second stage of the mapping exercise involved gathering tools to guide stakeholder and community engagement in QI initiatives, specifically for reproductive, maternal, newborn, child and adolescent health (RMNCAH). In December 2018, a request for tools was sent out via email to key informants from United Nations (UN) agencies, non‐governmental organizations (NGOs), listservs, authors of tools or programmes identified in the literature review and other known experts. Persons contacted were informed of the purpose of the mapping and asked to send formal documents used within their organization, or that they were familiar with, that met the inclusion criteria outlined below.

Collaboration was established with a concurrent call for tools to support country efforts for Integrated, People‐Centred Health Services, led by the WHO in collaboration with Johns Hopkins University. It was agreed that tools identified in the two exercises would be shared.

### 2.3 Inclusion and exclusion criteria

Tools identified through the two stages noted above were included in this mapping if they met the following criteria: (1) formal documents (handbooks, training guides, etc) that guide integration of stakeholder and community engagement in one or more of the seven phases of the Network's implementation framework (see Figure 2): establish leadership and stakeholder group; situational analysis; adapt standards of care; identify QI interventions; implementation of QI interventions; continuous measurement of quality and outcomes; and refinement of strategies; (2) focus on maternal, newborn and child and adolescent health, and/or family planning; and (3) published or revised after 2000. There were no restrictions on languages or geography.

We originally intended to include only materials related to RMNCAH; however, based on the large number of tools received and recognizing that processes of stakeholder and community engagement in other health and development areas could be useful and applicable to RMNCAH, we broadened the inclusion criteria. Additionally, the original call for tools requested documents published after 2010 to reflect resources that were developed based on more recent evidence; however, based on the materials and tools that were received through the mapping, we expanded this criterion to tools published after 2000 to ensure incorporation of relevant resources.

We excluded any tools that (1) only describe, outline or recommend an intervention but do not provide operational guidance on the QI implementation process; (2) were not relevant to stakeholder and community engagement in QI; or (3) were published before 2000.

### 2.4 Data extraction and synthesis

One author (JS) received and logged all tools in Excel. JS and AP reviewed and discussed all tools against inclusion and exclusion criteria and reached agreement on any materials received that were not clear. A third reviewer (BG) reviewed 20% of all identified items and any differences were resolved by discussion between all three reviewers. JS extracted article information in line with a predefined data extraction template (Data S1), in which characteristics were coded such as level of application (global or country/context‐specific tool), health area (RMNCAH, other health or non‐health) and phase of the QI process. AP reviewed the extraction tables. Key informants were contacted via email when clarifications were needed.

In August 2019, once all tools had been categorized, all authors and/or organizations of the included tools were contacted to request evaluation materials related to the specific tool, including materials, checklists and protocols used to evaluate the intervention, as well as evaluation reports and publications. The results of any evaluation would be valuable information for any future user of the tool.

## 3 RESULTS

**FIGURE 3:**
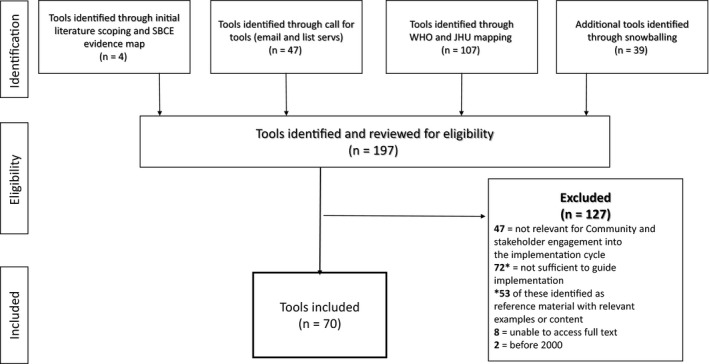
Flow diagram of the screening and selection process of received tools

The mapping yielded 197 documents that were screened for eligibility against our inclusion and exclusion criteria. Figure 3 outlines the searching and screening process. In total, we identified 70 tools to be included. Of the 127 excluded documents, 53 were categorized as reference material, in that they were not tools or implementation guidance documents, but their content could provide insight, instruction and explanation on how to engage stakeholders and communities into different phases of the implementation cycle. Tools have not been assessed for quality and are not endorsed by the WHO.

Data S1 contains details on the Included Tools and maps the tools to the seven phases of the QoC Network implementation framework. Data S2 contains details on the 53 reference materials, including reasons for exclusion and additional classifications for reference including whether the tool is relevant to general information on QI cycles (n = 23), QI process descriptions (n = 7), QI programme example (n = 13) or documents specific to participatory rural appraisal (n = 10).

### 3.1 Tool characteristics

Included tools were published between 2000 and 2019. The majority (70%) were published after 2010, primarily from 2013 to 2017; this is likely linked to our call that specifically requested materials developed after 2010. International NGOs were the most frequently cited authors of tools (n = 24), followed by government organizations (n = 13), UN agencies (n = 10), academic institutions or research centres (n = 9), development assistant agencies (n = 7) and bilateral development agencies (n = 5), namely the United States Agency for International Development and Deutsche Gesellschaft für Technische Zusammenarbeit. Only two national NGOs, one each from India and the United States, solely authored an included document. Professional organizations, coalitions and consortiums were the remaining authors. Approximately one third of the tools had multiple authors.

Most of the tools (48 of the 70) included were designed for global application and two focused on the Africa region. The remainder maintained a specific country focus, including Australia, Burundi, Germany, India, Mali, Morocco, New Zealand, Scotland, Sierra Leone, Spain, South Africa, the United Kingdom and the United States of America. Tools identified are predominantly written in English; three of the included tools are in French, and one tool is in Gujarati and was translated by the authoring organization, SAHAJ, India, upon request.

Twenty‐three tools specifically focused on reproductive, maternal, Newborn, Child and Adolescent Health (RMNCAH) (Table 1). Of these, all but eight tools addressed more than one RMNCAH topic, with six tools addressing two topics, and one tool addressing all topics within RMNCAH.^30^ Maternal health was addressed by the majority of tools (n = 14), followed by child health (n = 12), then reproductive (n = 10), newborn (n = 9) and adolescent health (n = 9), with one tool specifically addressing family planning. For RMNCAH tools, International NGOs were the most common author (n = 10), either solely or as lead, followed by UN bodies (n = 3), bilateral organizations (n = 3), governments (n = 2), consortiums (n = 2) with one each from local government, academia and development agencies.

**TABLE 1:** Characteristics of included tools focusing on Reproductive, Maternal, Newborn, Child and Adolescent Health topics

Tool	Target group for tool use	QI phases	R	M	N	C	A	FP	Specific focus on inequities/marginalized groups
Summary Reflection Guide on a Human Rights‐Based Approach to Health: Application to sexual and reproductive health, maternal health and under‐5 child health: Health Policy Makers	Health Policy Makers	1, 2, 3, 4, 5, 6, 7	x	x	x	x			Highlights need to include marginalized populations, but little/no guidance on how to engage with such groups
Summary Reflection Guide on a Human Rights‐Based Approach to Health: Application to sexual and reproductive health, maternal health and under‐5 child health: Health Workers	Health Workers	1, 4, 5, 6	x	x	x	x			Highlights need to include marginalized populations, but little/no guidance on how to engage with such groups
Partnership Defined Quality Manual Facilitation Guide and Manual & Monitoring and Evaluation Toolkit with Youth Annex^a^	Managers and health workers, teachers or community advocates	1, 2, 3, 4, 5, 6, 7		x	x	x	x		Highlights need to include marginalized populations, but little/no guidance on how to engage with such groups
Partnership Defined Quality for Youth: A Process Manual for Improving Reproductive Health Services Through Youth‐Provider Collaboration	Youth, health workers, implementers	1, 2, 3, 4, 5, 6, 7	x				x		Highlights need to include marginalized populations and some tools include guidance on how to support engagement with such group
Citizen Voice and Action Field Guide (2013) & The Citizen Voice and Action Guidance Notes (2016)^a^	Implementers	1, 2, 4, 5, 6		x	x	x			Highlights need to include marginalized populations, but little/no guidance on how to engage with such groups
Working with individuals, families and communities to improve maternal and newborn health: a toolkit for implementation	Government actors	1, 2, 3, 4, 5, 6, 7		x	x				Highlights need to include marginalized populations and some tools include guidance on how to support engagement with such groups
Facility management committees (FMCs) Operational guidelines & Training Manuals and Tools	District Health Management Teams and Implementing Partners	1, 2, 3, 4, 5, 6		x	x	x			Highlights the need for representation from populations with special needs on FMCs, but does not guide on how to engage such groups
Advancing Partners and Communities: Strengthening Health Services as part of the Post‐Ebola Transition in Sierra Leone, Community Engagement and Implementation Strategy and Toolkit	District Health Management Teams and Implementing Partners	1, 2, 3, 4, 5, 6	x	x	x	x			Highlights need to include marginalized populations and some tools include guidance on how to support engagement with such group
Healthy Mother Tool: Community Promotion for Maternal Health Care & Report Card on Maternal Health Services in Two Blocks of Dahod and Panchmahal Districts of Gujarat	Health Workers	2, 6		x					No specific focus on inequities.
Developing a country RMNCH scorecard: Facilitator’s guide	Ministry of Health	1, 2, 4, 5, 6	x	x	x	x			No specific focus on inequities.
Facilitator's module for Civic Education on Governance in Sexual and Reproductive Health and Rights and Family Planning 2014	Implementers	1, 4, 5, 6	x					x	Highlights need to include marginalized populations, but little/no guidance on how to engage with such groups
Handbook for Budget Analysis and Tracking in Advocacy Projects	Implementers, Civil Society	1, 2, 6	x						No specific focus on inequities.
Handbook for Political Analysis and Mapping	Implementers, Civil Society	1, 2, 4, 5	x						No specific focus on inequities.
A Health Workers Training Manual: Community Engagement for Quality Care^a^	Health Workers	1, 2, 3, 4, 5, 6, 7		x					Highlights importance, but little guidance on inclusion
A guide for advocating for respectful maternity care	Advocates	1, 2, 3, 4, 5, 6, 7		x					Highlights need to include marginalized populations and some tools include guidance on how to support engagement with such group
Demanding Accountability for Women's and Children's and Adolescent's Health: Citizen's Hearing Toolkit 2016	Implementers	1, 2, 6	x	x	x	x	x		No specific focus on inequities.
Children’s Participation in the Analysis, Planning and Design of Programmes: A guide for Save the Children staff & Toolkit for Monitoring and Evaluating Children’s participation	Implementers	1, 2, 3, 4, 5, 6, 7				x	x		Highlights need to include marginalized populations and some tools include guidance on how to support engagement with such group
The Program Assessment Guide: An Approach for Structuring Contextual Knowledge and Experience to Improve the Design, Delivery, and Effectiveness of Nutrition Interventions	Implementers	1, 2, 3, 4, 5, 6		x		x			Highlights need to include marginalized populations and some tools include guidance on how to support engagement with such group
Engaging your community, A Toolkit for partnership, collaboration and action	Implementers	1, 2	x				x		Highlights need to include marginalized populations and some tools include guidance on how to support engagement with such group
Toolkit for monitoring and evaluating gender‐based violence interventions along the relief to development continuum	Implementers	1, 6		x		x	x		Highlights need to include marginalized populations and some tools include guidance on how to support engagement with such group
A Handbook for Community Participatory Assessments	Government actors	1, 2, 5, 6				x	x		No specific focus on inequities.
Youth Participation Guide: Assessment, Planning, and Implementation	Implementers Youth	1, 2, 3, 4, 5, 6					x		Highlights need to include marginalized populations and some tools include guidance on how to support engagement with such group
The UNICEF Project Cycle Booklets 3 & 4 Three: Adolescent Participation and the Project Cycle Four: Tools for Adolescent and Youth Participation	Implementers, Policy Makers, Teachers,	1, 2, 3, 4, 5, 6, 7					x		Highlights need to include marginalized populations and some tools include guidance on how to support engagement with such group.
		Total:	10	14	9	12	9	1	

^a^ Tool covers various health issues and provides examples of MNCH topics within the guide, but was not designed solely for MNCH.

The large majority of the RMNCAH tools were intended for use by NGO programme staff and health workers or managers to support the engagement of stakeholders and communities. Two tools were intended for use by stakeholders and communities themselves (‘youth’ in both cases), either alone or in conjunction with implementers.^31^, ^32^ Seventeen of the 23 tools noted the need to include marginalized populations within engagement processes, but only 10 of these had specific elements within their tools to support engagement activities with these populations. Often, these included one or two additional considerations for marginalized populations but did not contain detailed guidance for ensuring such involvement.

The remainder of the tools covered a wide range of topics. Nineteen were designed for other general health topics and the health‐care system, including for facility management committees,^33^ demand and use of data^34^ and systems analysis using Group Model Building.^35^ Twenty‐six tools addressed non‐health subjects. Fifteen of these are general participatory tools to be adapted across multiple sectors, issues and organizations, including health. For example, CARE’s Community Score Card^36^ is a tool designed to be used in any sector (ie health, education and agriculture), at any level (local, regional, country, etc) and by a variety of stakeholders (NGO, local government officials, health workers, educators, etc). Evaluation materials were requested from authors of the included tools, with evaluation materials identified for 15 of the 70 tools.

### 3.2 Tools for stakeholder and community engagement across the QI implementation framework

**FIGURE 4:**
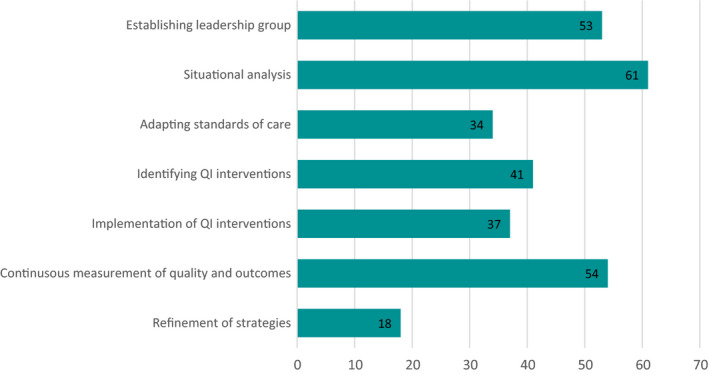
Tools across quality improvement implementation phases

A main interest of this exercise was to map tools to the different phases in the Network QI implementation framework (Figure 2). Figure 4 displays the number of tools related to each phase of the implementation framework. Fifteen tools covered all seven phases of the QI cycle. For the RMNCAH tools, all provided guidance on more than one phase of QI implementation with eight tools covering all seven phases of QI implementation.

For all included tools, most provide guidance on establishing leadership groups (phase 1), situational analysis (phase 2) and continuous measurement of quality and outcomes (phase 6) and related initiatives, at 76%, 87% and 77%, respectively. Less guidance was identified on involving stakeholders and community members into adapting standards of care (phase 3), identifying QI interventions (phase 4) and implementation of QI interventions (phase 5). Few tools were identified on stakeholder and community engagement for refinement of strategies (phase 7); of the only 15 tools that did cover phase 7, 12 of these covered all of the phases within the QI framework. Phases 1, 2 and 6 were the only ones to occur independently, at two, three and two instances, respectively. Phase 2 and 6 frequently occurred together, with eight tools covering only these two domains. Data S1 maps all included tools to the phases.

There is a wide variation in how each of the included tools addresses the phases of QI implementation. Some documents, such as Save the Children's ‘The Spider Tool’^37^ for planning and self‐assessment of child‐led initiatives, or Community Scorecards^36^ focus on a specific activity/resource that can be applied to one or more phases. Others include more implementation approaches for stakeholder and community engagement that can be undertaken to support QI across multiple phases, like facility management committees,^33^ or Community Health Committees.^38^ Others still tackle broad concepts within QI, such as partnerships building and/or maintaining,^39^, ^40^, ^41^ data use or evaluation^34^, ^42^ and, more broadly, participation across the project cycle,^32^, ^43^, ^44^ including numerous approaches that can be undertaken. The latter type typically covers more phases of QI implementation, compared to more discrete activities/resources.

## 4 DISCUSSION

This mapping indicates that many tools are already available on stakeholder and community engagement in aspects of QI and highlights the importance of collecting available resources within this field to build upon and avoid duplication. As the tools included in this exercise have not been assessed for quality, it is hard to conclude that new or more tools are not needed. However, the mapping demonstrates the importance for programmes to take a comprehensive approach to stakeholder and community engagement (addressing all seven phases), to adapt the tools and implementation to specific contexts and to include an evaluation of the effectiveness of the tools. Included tools are available through an online portal on the WHO website (available at: https://www.who.int/activities/tools‐to‐support‐the‐integration‐of‐stakeholder‐and‐community‐engagement‐in‐quality‐of‐care‐initiatives‐for‐maternal‐newborn‐and‐child‐health *)*. The portal allows users to identify tools through filters based on the characteristics outlined above.

There appears to be uneven guidance per activity across the QI implementation framework. Topics that relate to consultation with stakeholders and communities (such as phases 1 and 2) and monitoring (phase 6) were more frequently covered, but stakeholder and community involvement in intervention development, implementation and evaluation was less so. This differs from findings in a systematic review by George et al^6^ that found that communities mainly participated during the implementation of health interventions, and less in design phases, problem and situational analyses, or in managing resources, monitoring or evaluation. Additional discussion among practitioners to understand levels and intensity of engagement in the phases of QI initiatives would be fruitful.

The authors noted a lack of evaluation of the included tools. Of those that did conduct an evaluation (n = 15), the majority consisted of internal reports (ie endline studies, lessons learned documents) with only five evaluations involving discrete research pieces subject to peer review. Of the 15 tools evaluated, the tools themselves were not often the subject of the study but a component reported within a wider evaluation. Evaluating complex approaches, such as QI initiatives and tools, is no easy feat, and globally, there exists a need to understand how to strengthen evaluation and research methodologies to better address this complexity.^45^ While quality measures (e.g. patient‐provider communication) and health outcome measures (e.g. improved care seeking) are important, other social impacts of stakeholder and community engagement interventions (e.g. increased rapport and trust between communities and health services) coupled with longer‐term social transformations (e.g. social cohesion and community empowerment) and effects on equity would also be important to capture.^46^

Most tools were targeted for global purposes and were developed by international NGOs. A next step for the Network would be to work with partner organizations to understand processes for local adaptation and translation of the predominantly English materials. The country‐specific tools can also be reviewed and adapted to other contexts; these tools may offer valuable insights for regional and country‐specific issues and goals.

Similarly, many of the tools did not have a specific topic focus (i.e. RMNCAH) but offered a broader guidance on stakeholder and community engagement in QI processes. Of the 70 included tools, 23 addressed RMNCAH, primarily designed by international NGOs or a UN agency for global implementation. This trend in results brings to question if health area specificity is essential or if context‐specific details can be captured in adaptation. For example, ‘Working with individuals, families and communities to improve maternal and newborn health: a toolkit for implementation’^47^ guides district governments on integrating health promotion interventions through a maternal and newborn health lens, addressing how to amplify the voice of women and other stakeholders (i.e. local authorities, traditional birth attendants), and highlights barriers to care seeking for maternal and newborn health. Such content‐specific insight may be lost in more generic stakeholder and community engagement in QI tools.

We speculate that tools specific to every health topic may not be required; rather, generic principles and approaches, with examples and cases of specific sectors or health content, may be sufficient. Generic guidance should support the ability to identify strategic and practical objectives of stakeholder and community engagement at different levels of the health system, to conduct stakeholder mapping and analysis for specific QI goals and to learn to adapt tools and processes to the local context. This may help to avoid a rigid, mechanistic or ad hoc approach to applying stakeholder and community engagement, even when considering developing at a larger scale, for instance in country programmes.

Ultimately, the success of stakeholder and community engagement initiatives will depend on broader programme and contextual factors in which initiatives are occurring.^48^ Tools need to be considered within a wider process of QI and health system development, and the many factors that influence stakeholder and community engagement will require consideration. This is echoed by authors advocating for improving capability of stakeholders, communities and health actors ensuring linkages with health system, supportive policy and funding environments^7^, ^49^ and understanding of population‐level motivation and limitations.^48^

It was beyond the scope of this mapping to assess whether included tools were informed by underlying approaches, such as Human Rights‐Based Approach, or integrated interculturality, intersectionality and/or gender considerations in their design and/or implementation. However, to support adaptation, countries or organizations are encouraged to consider such aspects of a tool's development and focus, ensuring alignment with the programme's goals to support more contextually informed implementation.

Regardless of the QI phase in which stakeholder and community engagement is initiated, it seems to be particularly important to engage in early interaction and conversation with stakeholders and communities^50^ to build support and trust and align with broader community needs.^6^ While this does not come out clearly within the framework phases, many tools and the literature emphasize the importance of establishing an environment of respect and trust, collaboration and partnerships, preparing participants to value the knowledge different partners can bring to the discussions, to build relationships and articulate expectations and roles.^7^

Notably, our findings show that most tools include some form of participatory situational analysis involving stakeholders and communities. Moreover, several tools include a strong community mobilization and advocacy component to raise communities’ awareness on the importance of participating in the QI process and demanding improvements in quality for all. For example, White Ribbon Alliance's tool, ‘A guide for advocating for respectful maternity care’,^51^ presents the different elements of advocacy and community mobilization with respect to demanding respectful maternity care. Furthermore, the ‘Citizens’ Hearing Toolkit’^30^ instructs communities on public hearings in which service users, community members and other stakeholders discuss gaps and weaknesses in the provision and experiences of care in order to increase access to quality RMNCAH services. Other tools include a component of strengthening managers’ skills to communicate with different partners or offer guidance to building strong, effective partnerships like the ‘Partnership Culture Navigator’^39^ or ‘Partnership Defined Quality’.^52^

It is likely that these essential, more nuanced aspects of stakeholder and community engagement are often difficult to capture in tools. Aligned to this, the Network suggests the way forward for countries is to stimulate Leadership, Action, Learning and Accountability.^28^ Learning from those who have experience, learning within facilities and communities, sharing the learning among different sites in countries and between national, district and local levels and then between countries, will be essential for integrating stakeholder and community engagement in QI initiatives in a meaningful and effective way. Experience shows that once a QI team gains practice, they can then expand from a singular focus on one health area to apply these skills to other health topic areas.^47^

Stakeholder and community engagement should become part of the routine functioning of health systems; mechanisms and relationships established can then be leveraged for other purposes or at times of urgent need, for instance in responding to infectious disease emergencies. The established systems and relationships for engagement can support wider health systems strengthening^53^ and responsiveness.^54^, ^55^ Meaningful stakeholder and community engagement for RMNCAH QI therefore not only supports quality of care, but can have positive repercussions on the wider health system, while also supporting individuals to participate in the care they receive.

### 4.1 Limitations

While we utilized several methods to search and identify relevant tools, our strategy may have resulted in missed items. This limitation may have been reduced by conducting a parallel structured systematic review, which supported the identification of additional tools or snowballing sources. We were reliant on email and listserv responses and may not have reached all relevant individuals and organizations. While we employed multiple levels of screening and had two screeners, the complexity of these tools makes straightforward inclusion/exclusion difficult; for example, some relevant resources may have been excluded if they did not align to the seven phases. There were very few tools that were developed by national or local organizations as these resources may have been harder to identify through our searching strategy. An important next step for countries within the Network will be to expand this inventory by gathering the tools within their own contexts.

## 5 CONCLUSION

Given the considerable number of tools available, developing more tools may not be necessary at this time. WHO and UNICEF have gone forward developing a module to outline the important concepts of stakeholder and community engagement while making links to the available resources identified through this mapping. There is a need to understand local adaption of global or context‐specific guidance and how tools on different topics can be applied and adapted to the particularities of specific health topics (i.e. RMNCAH). Future work should support the more rigorous evaluation of such tools and determine how to expand measures of success beyond access and health outcomes. The capturing of lessons learned and impact on building trust among the different stakeholders and other longer‐term social impacts is essential. The variation within tools and the overlapping guidance found within the included resources confirm that stakeholder and community engagement cannot be captured in a ‘one‐size‐fits‐all’ formula. Countries and programmes will need to explore available tools and adapt these to their relevant needs and specific context. However, this field may benefit more from understanding such initiatives and supporting the tailoring and adaptations of existing materials, as opposed to developing more stand‐alone resources.

## 6 DISCLAIMER

The authors alone are responsible for the views expressed in this article and they do not necessarily represent the views, decisions or policies of the institutions with which they are affiliated.

## CONFLICT OF INTEREST

None declared.

## AUTHOR CONTRIBUTIONS

AP, BG and JS designed the methodology. BG conducted the initial literature search and literature scoping. For the mapping exercise, JS conducted the call for tools, JS screened the literature, JS and AP conducted the screening of resources, and BG reviewed the screening results. JS wrote up a first synthesis of included tools. All authors prepared and approved the manuscript.

## Supporting information

Data S1

Data S2

## Data Availability

The data that support the findings of this study are available in the supplementary material of this article.
