# Supplementary material for: A mapping and synthesis of tools for stakeholder and community engagement in quality improvement initiatives for reproductive, maternal, newborn, child and adolescent health
Source: Health Expect. 2021 Apr 1;24(3):744–56. doi: 10.1111/hex.13237 (PMC8235899; doi:10.1111/hex.13237)
Supplement: Supplementary file 2 — Data S2 [file HEX-24-744-s002.docx]

| **Reference Material Classification** |
| --- |
| Numerous documents were identified that provided valuable insight and guidance into stakeholder and community engagement for quality of care but did not contain sufficient detail to guide implementation within the specific QI cycle steps and were thus excluded. 53 documents were classified as reference materials and were further categorized. 23 documents provided general information to elements of the QI cycle: these documents often described different stages in the cycle, provided rationale for SCE, and highlighted key principles of SCE into the QI cycle without providing technical or methodological guidance. Seven reference documents included process descriptions, including examples of interventions and descriptions of different phases in the QI cycle. 13 reference materials gave program examples. Although these documents reference specific interventions, these were merely descriptions, such as case studies, without implementation guidance. Finally, 10 documents discussed Participatory Rural Appraisal (PRA) and approaches. |
| General Information on QI Cycle |
| 1. Department of Health and Human Services, USA. NIH Publication No. 11-7782. DJ, Aguilar-Gaxiola, S, Michener JL. Principles of Community Engagement: Second Edition. 2011 https://www.atsdr.cdc.gov/communityengagement/index.html. |
| 2. Appel K, Buckingham E, Jodoin K, Roth D. Participatory Learning and Action Toolkit: For application in BSR’s Global Programs. 2012. https://herproject.org/files/toolkits/HERproject-Participatory-Learning.pdf. |
| 3. Center for Community Health and Evaluation and Human Impact Partners. Community Participation in Health Impact Assessments: A National Evaluation. 2015. https://humanimpact.org/wp-content/uploads/2018/10/Full-report_Community-Participation-in-HIA-Evaluation.pdf. |
| 4. Cook P, Blanchet-Cohen N, Hart S. Children as Partners: Child Participation promoting social change. 2004. https://resourcecentre.savethechildren.net/node/2352/pdf/2352.pdf. |
| 5. Cramer ME, Atwood JR, Stoner JA. Measuring community coalition effectiveness using the ICE instrument. *Public Health Nurs*. 2006;23(1):74-87. doi:10.1111/j.0737-1209.2006.230111. |
| 6. Halper E. Moving on: Effective management for partnership transition, transformations and exits. 2009. https://thepartneringinitiative.org/publications/toolbook-series/moving-on/. |
| 7. Maack JN. Scenario Analysis: A Tool for Task Managers. Social Analysis Selected Tools and Techniques. 2001;36. http://siteresources.worldbank.org/INTCDD/Resources/SAtools.pdf#page=68. |
| 8. Martens M, Toonen J, van der Waal B. Monitoring quality of care and accountability mechanisms at the district level: The potential role of the National Health Insurance Scheme in Ghana. 2011. |
| 9. McCarthy S, O’Raghallaigh P, Woodworth S, Lim YL, Kenny LC, Adam F. An integrated patient journey mapping tool for embedding quality in healthcare service reform. *Journal of Decision Systems*. 2016;25(sup1):354-368. doi:10.1080/12460125.2016.1187394 |
| 10. McManus S, Tennyson R. Talking the Walk: Talking the Walk. A communication manual for partnership practitioners 2008. https://thepartneringinitiative.org/publications/toolbook-series/talking-the-walk/. |
| 11. Russel N, Igras S, Johri N, Kuoh H, Pavin M, Wickstrom J. The Active Community Engagement Continuum. 2008. https://pdf.usaid.gov/pdf_docs/pnadm497.pdf. |
| 12. Sheedy A, MacKinnon MP, Pitre S, Watling J. Handbook on Citizen Engagement: Beyond Consultation. March 2008. https://ccednet-rcdec.ca/sites/ccednet-rcdec.ca/files/handbook_on_citizen_engagement.pdf. |
| 13. Stott L. The Partnering with Governments Navigator. 2011. https://thepartneringinitiative.org/publications/toolbook-series/the-partnering-with-governments-navigator/ |
| 14. Tennyson R. The Brokering Guidebook. Navigating effective sustainable development partnerships 2005. https://thepartneringinitiative.org/publications/toolbook-series/the-brokering-guidebook/. |
| 15. Walker T. The 5Rs Framework in the Program Cycle. USAID Learning Lab. https://usaidlearninglab.org/library/5rs-framework-program-cycle. Published October 25, 2016. |
| 16. Warren L. Welcoming & Inclusive Communities Toolkit: Templates and tools for Alberta’s municipalities. 2014. http://citiesofmigration.ca/wp-content/uploads/2017/10/AUMA-Welcoming-and-Inclusive-Communities-Toolkit.pdf. |
| 17. HEPS – Uganda. Community Training Guide on Rights Based Family Planning. 2015. https://www.slideshare.net/Hepsuganda/reproductive-health-training-manual-heps-uganda. |
| 18. Ministry of Health and Family Welfare, Government of India Quality Standards for Urban Primary Health Centre December 2015. December 2015. http://tripuranrhm.gov.in/QA/Guideline/QualityStandardsforUrbanPrimaryHealthCentre.pdf. |
| 19. Centre for Disease Prevention and Control. A Practitioner’s Guide for Advancing Health Equity: Community Strategies for Preventing Chronic Disease. 2019 https://www.cdc.gov/nccdphp/dnpao/state-local-programs/health-equity-guide/index.htm. |
| 20. UNICEF. Adolescent development and participation. 2010. https://www.unicef.org/adolescence. |
| 21. National Network of Libraries and Medicine, NIH. Tools and Resources for Community Assessment. nnlm.gov. https://nnlm.gov/neo/guides/tools-and-resources/community-assessment. |
| 22. Institute of Development Studies (IDS). Participatory Methods. participatorymethods.org. https://www.participatorymethods.org/page/about-participatory-methods. |
| 23.  Community Health and Development, University of Kansas. The Community ToolBox: Tools to change our world. ctb.ku.edu. <https://ctb.ku.edu/en>. |
| QI Process Descriptions |
| 1. Jones E, Mafani M, eds. Analysis, Design and Planning Tool (ADAPT) for Health and Nutrition Guidebook. 2014. |
| 2. McGinn E, Lipsky A. Social Accountability: A Primer for Civil Society Organizations Working in Family Planning and Reproductive Health. 2015. https://www.healthpolicyproject.com/pubs/449_PSocialAccountabilityReportFINALEC.pdf. |
| 3. Roma S, Levine C. Saving Newborn Lives: Champions Toolkit. 2016. http://www.healthynewbornnetwork.org/hnn-content/uploads/SNL-Champions-Toolkit-Final-May16.pdf. |
| 4. James, A.J. Monitoring Impact. In: Enhancing Ownership and Sustainability: A Resource Book on Participation. International Fund for Agricultural Development (IFAD), Asian NGO Coalition for Agrarian Reform and Rural Development (ANGOC) and International Institute of Rural Reconstruction (IIRR); 2001:206-262. http://www.managingforimpact.org/sites/default/files/resource/enhancing_ownership_and_sustainability_part4.pdf. |
| 5. Management Sciences for Health. Community-based participation and initiatives. In: *MDS-3: Managing Access to Medicines and Health Technologies (Third Edition)*. 2012:31.1-31.17. https://www.msh.org/sites/msh.org/files/mds3-ch31-community-mar2012.pdf |
| 6. Maternal and Perinatal Death Surveillance and Response Guidelines. August 2017. http://health.go.ug/content/maternal-and-perinatal-death-surveillance-and-response-guidelines-aug-2017. |
| 7. Center for Disease Prevention and Control. Community Health Assessments and Health Improvement Plans. 2019. <https://www.cdc.gov/publichealthgateway/cha/plan.html>. |
| QI Program Examples |
| 1.  Evans, K.; Velarde, S.J.; Prieto, R.; Rao, S.N.; Sertzen, S.; Davila, K.; Cronkleton, P.; de Jong, W. *Field Guide to the Future: Four Ways for Communities to Think Ahead*. (Bennett E, Zurek M, eds.). Nairobi: Center for International Forestry Research (CIFOR), ASB, World Agroforestry Centre; 2006. https://www.cifor.org/library/2137 |
| 2. Ho A, Coates P. Citizen-Initiated Performance Assessment: The Initial Iowa Experience. *Public Performance & Management Review*. 2004;27(3):29-50. doi:10.1080/15309576.2004.11051800 |
| 3. Bintz, M. How Can Asset Mapping Improve Community Health? ihi.org. http://www.ihi.org:80/education/IHIOpenSchool/resources/Pages/Activities/Bintz-AssetMapping.aspx. |
| 4. National Institute for Health and Care Excellence (NIHR). Community engagement: improving health and wellbeing and reducing health inequalities. 2016. https://www.nice.org.uk/guidance/ng44/resources/community-engagement-improving-health-and-wellbeing-and-reducing-health-inequalities-pdf-1837452829381. |
| 5. Olivares M. Geographic Information Systems at Yale: Community Mapping Resources. guides.library.yale.edu. https://guides.library.yale.edu/c.php?g=295854&p=1972667. |
| 6. The Community Engagement Network, Resource and Regional Services Division, Victorian Government Department of Sustainability and Environment, Melbourne, Australia. Book 3: The Engagement Toolkit Effective Engagement: Building Relationships with Community and Other Stakeholders. 2005. <https://www.betterevaluation.org/sites/default/files/effective%20engagement%20book%203.pdf>. |
| 7. USAID. Promoting Accountability For Safe Motherhood: The White Ribbon Alliance’s Social Watch Approach. 2010. http://www.healthpolicyplus.com/archive/ns/pubs/hpi/1282_1_Social_Watch_WRA_HPI_FINAL_acc.pdf. |
| 8. Enfants du Monde. Guide D’orientation Pour La Rencontre De Plaidoyer Avec Les Leaders Communautaires Sur L’implication Des Hommes Dans La Sante Maternelle Et Neonatale |
| 9.  The World Bank. Community-Driven Development Toolkit: Governance and Accountability Dimensions Module 1.2. 2019. <https://www.worldbank.org/en/topic/communitydrivendevelopment/publication/community-driven-development-toolkit-governance-and-accountability-dimensions>. |
| 10. National Collaborating Centre for Methods and Tools (NCCMT), McMaster University. Knowledge Translation Methods and Tools for Public Health. https://www.nccmt.ca/knowledge-repositories/search. |
| 11. MYRADA. Participatory Mapping & Modelling –https://myrada.org/participatory-mapping-modelling/. |
| 12.Stevens, K. Rich Pictures. 2016. https://www.betterevaluation.org/evaluation-options/richpictures. |
| 13. Federal Democratic Republic of Ethiopia, Ministry of Health. National Reproductive Health Strategy 206-2020. 2016. <http://corhaethiopia.org/wp-content/uploads/2016/08/RH-strategy-2016.pdf>. |
| Participatory Rural Appraisal |
| 1. Geilfus F. 80 Tools for Participatory Development: Appraisal, Planning, Follow-up and Evaluation. San Jose, Costa Rica: Inter-American Institute for Cooperation on Agriculture (IICA); 2008. http://repiica.iica.int/docs/B1013I/B1013I.pdf. |
| 2. Lynam T. Participatory Systems Analysis: an Introductory Guide. 2001. <https://opendocs.ids.ac.uk/opendocs/bitstream/handle/123456789/6231/Lynam,%20%20Tim.%20%20IES%20%20Special%20Report%20no.%2022..pdf;jsessionid=C3ADD06C2C9189C26E96717DBD38D344?sequence=1>. |
| 3. National Institute of Rural Development & Panchayati Raj. Participatory Rural Appraisal (PRA). <http://www.nird.org.in/nird_docs/gpdp/pra.pdf> |
| 4. Narayanasamy N. *Participatory Rural Appraisal: Principles, Methods and Application*. New Delhi; 2009. doi:10.4135/9788132108382. http://sk.sagepub.com/books/participatory-rural-appraisal |
| 5. Pepall E, James R, Dantas J. Guidelines for Conducting Rapid Participatory Appraisals of Community Health Needs in Developing Countries: Experience from Tulikup, Bali. *Asia-Pacific journal of public health / Asia-Pacific Academic Consortium for Public Health*. 2006;18:42-48. doi:10.1177/10105395060180030801. https://journals.sagepub.com/doi/pdf/10.1177/10105395060180030801?casa_token=j4OAck04YuEAAAAA:OPbSVe1gpCYgWEzCfdW_txlgMgix6lz_ksEO97srkd48SrD46DskJmsvDdLjiKy8pUmw9Utl4si |
| 6. World Food Programme. *Participatory Techniques and Tools: A WFP Guide*. 2001. https://toolkit.ineesite.org/resources/ineecms/uploads/1033/Participatory_Techniques_EN.pdf |
| 7. Department of Planning and Investment (DPI), Viet Nam & Quang Ngai Rural Development Program (RUDEP). Participatory Rural Appraisal Manual. 2007. https://himachal.nic.in/WriteReadData/l892s/15_l892s/1499233748.pdf. |
| 8.  Catalytic Communities (CatComm). Community Mapping through Transect Walks. 2013 https://catcomm.org/transect-walk/. |
| 9. Chatty, D., Baas, S., and Fleig, A.. Module II: Introducing Participatory Approaches, Methods and Tools. 2003. http://www.fao.org/3/ad424e/ad424e03.htm. |
| 10.  Lienert, J. Participatory Mapping for Decision Making. https://sswm.info/planning-and-programming/decision-making/deciding-community/participatory-mapping-for-decision-making.  11. See reference 22 in the “General Information” section, which also discusses PRA. |
